# Mental health and attitudes toward suicide amongst individuals with gender dysphoria in Iran

**DOI:** 10.3389/fpsyt.2024.1443638

**Published:** 2024-12-09

**Authors:** Maryam Assareh, Vahid Rashedi, Mehrdad Eftekhar Ardebili, Razieh Salehian, Mohammadreza Shalbafan

**Affiliations:** ^1^ Mental Health Research Center, Psychosocial Health Research Institute, Department of Psychiatry, School of Medicine, Iran University of Medical Sciences, Tehran, Iran; ^2^ Iranian Research Center on Aging, Department of Aging, University of Social Welfare and Rehabilitation Sciences, Tehran, Iran; ^3^ Central Queensland Mental Health, Alcohol, and Other Drugs Services, Rockhampton, QLD, Australia

**Keywords:** gender dysphoria, suicide, mental health, depression, attitudes, Iran

## Abstract

**Introduction:**

Gender dysphoria (GD) is associated with profound mental health challenges, including heightened suicide risk. This study aimed to explore the mental health status and attitudes toward suicide among individuals with GD in Iran.

**Methods:**

A comprehensive assessment was conducted using three validated tools: the General Health Questionnaire (GHQ-28) to gauge emotional distress and psychological well-being, the Beck Depression Inventory (BDI-II) to evaluate the severity of depressive symptoms, and the Predicament Questionnaire (PQ) to measure attitudes toward suicide in the context of social crises.

**Results:**

Among the 78 participants, 43 (55.1%) were identified as experiencing significant mental health issues, while 21 (26.9%) were found to suffer from severe depression. The average PQ score was 57.92, which aligns with findings from similar populations in previous studies, highlighting a concerning trend in suicide-related attitudes. Further statistical analysis revealed significant correlations between psychological distress (GHQ-28 scores) and attitudes toward suicide (PQ scores), as well as between depressive symptoms (BDI-II scores) and suicide-related attitudes.

**Conclusions:**

These findings indicate a pervasive prevalence of psychological disorders, particularly depression, within this group, and underscore the strong association between depressive states and suicidal ideation. The study underscores the urgent need for targeted mental health interventions and comprehensive healthcare policies tailored to the specific needs of individuals with GD in Iran. Given the substantial mental health burden observed, particularly the risk of suicide, this research highlights the critical importance of integrating mental health care into broader support systems for this population.

## 1 Introduction

Gender identity minorities represent one of the most vulnerable groups with a significantly elevated risk of suicide (1). Gender identity encompasses an individual’s deeply held sense of being male, female, or another identity outside the traditional binary, such as non-binary or genderqueer (2). Based on the Diagnostic and Statistical Manual of Mental Disorders, Fifth Edition (DSM-5), gender dysphoria (GD) is defined as a pronounced incongruence between an individual’s experienced or expressed gender and their birth-assigned sex. This incongruence often brings substantial distress or impairment, particularly when societal or personal expectations do not align with one’s authentic gender experience (3). Individuals with GD encounter numerous risk factors, including interpersonal conflict, crises, exposure to violence, substance abuse, social isolation, and frequent experiences of discrimination. Each of these challenges intensifies their vulnerability, creating a uniquely challenging environment for mental well-being (4). Current conservative estimates indicate that individuals with GD represent approximately 0.1% to 0.5% of the global population, highlighting the significant yet often overlooked prevalence of this group worldwide (5). In Iran, the prevalence of GD is estimated to be 1.46 per 100,000 people, with evidence indicating an upward trend (6, 7).

Research on suicide within the GD population remains limited, leaving critical gaps in our knowledge and understanding of the factors that contribute to their heightened vulnerability. Studies indicate that individuals with GD experience significantly higher rates of depression than the general population (6, 8). This psychological burden, stemming from societal stigma, discrimination, and internal conflict related to their gender identity, often leads to heightened emotional distress (4). In Iran, individuals with GD encounter significantly greater challenges compared to their counterparts in Western countries, largely due to cultural, societal, and religious factors. These barriers not only exacerbate the stigma and discrimination they face but also severely limit their access to appropriate healthcare and social support. As a result, the psychological distress experienced by this group is often intensified. Moreover, research on GD in Iran remains scarce, with few studies dedicated to understanding the unique struggles and mental health needs of this population (6, 8). In the United States, a study by Wilton et al. (2018) reported a 6.36% prevalence of suicidal ideation among these individuals and other sexual minorities (4). By contrast, in a study carried out by Arianmehr et al. (2023), it was estimated that 83% of the Iranian population with GD experience suicidal ideation (8). Recent findings also indicates that those with GD who pursue medical treatment or hormone therapy attempt suicide between one to three times during their lifetimes which is a significant risk factor for suicide-related mortality (9).

While the predominant focus of previous studies on suicidal ideation and past suicide attempts among individuals with GD, there is a growing body of evidence suggesting that attitudes toward suicide may serve as a more accurate predictor of future suicide-related outcomes (10, 11). Understanding these attitudes is crucial, as they reflect the complex interplay of beliefs, emotions, and experiences that shape an individual’s relationship with self-harm and suicidal thoughts. By shifting the focus from merely assessing suicidal ideation to exploring the underlying attitudes, we may gain deeper insights into the factors that contribute to the risk of suicide, ultimately paving the way for more effective prevention strategies tailored to this vulnerable population (12). This study was designed to first to explore the mental health states and attitude towards suicide of people with GD, and secondly to assess how attitude towards suicide relates with depression and overall mental health of people with GD referred to the Tehran Psychiatric Institute, one of the leading referral centers in Iran.

## 2 Materials and methods

### 2.1 Study design and setting

In this cross-sectional study, data was collected from October 2021 to September 2022 from patients diagnosed with GD and referred to Tehran Psychiatric Institute, affiliated with the Iran University of Medical Sciences in Tehran, Iran. In Iran, individuals diagnosed with GD are required to undergo a series of psychiatric evaluations to qualify for gender-affirming surgery. These assessments are necessary before they are legally permitted to start the process of changing their gender. Therefore, all participants in our study completed the necessary forms before undergoing any gender-affirming treatments. All eligible participants who consented to join the study were included to ensure the sample accurately reflected the population of interest.

A medical intern from our team was present at Tehran Psychiatric Institute on the days selected for the psychiatric evaluation of individuals with GD. The researcher provided information about the study’s purpose and procedure, clarified that the study was independent of their screening process for surgery, and ensured confidentiality of responses. Only participants who agreed to join the study received the questionnaires, and the intern remained available to clarify any questions about the items.

### 2.2 Participants

The eligibility criteria required participants to have a diagnosis of GD, as defined by the DSM-5, which was confirmed by at least two independent psychiatrists. Additionally, participants needed to be over 18 years of age and had not yet undergone any gender-affirming surgery. Individuals were excluded if they were diagnosed with major psychiatric disorders such as schizophrenia or bipolar disorder, based on comprehensive psychiatric assessments.

### 2.3 Questionnaires

Data collection was conducted using the Persian versions of three questionnaires: The General Health Questionnaire (GHQ-28), the Beck Depression Inventory (BDI-II), and the Predicament Questionnaire (PQ).

#### 2.3.1 GHQ-28

Designed by Goldberg and Hillier, GHQ-28 evaluates emotional stress and psychological state of well-being over the past few weeks through 28 items rated on a 4-point Likert scale across four subscales: somatic symptoms, anxiety and insomnia, social dysfunction, and depression (13). The minimum score is 0 and the maximum is 84 with higher scores indicating higher levels of distress. GHQ-28 has been translated into Persian by Nazifi et al. and has been validated for the Iranian population (7). Ebrahimi et al. reported a Cronbach’s alpha coefficient of 0.97 for this questionnaire, with a cut-off score of 24 for suspected mental (14).

#### 2.3.2 BDI-II

BDI-II is one of the most common questionnaires for assessing behavioral symptoms of depression. It was first designed by Beck in 1961 (15). This questionnaire consists of 21 items with scores of 0 to 3. Scores higher than 30 indicate severe depression, 17 to 29 moderate depression, and 10 to 16 mild depression. Ghasemzadeh et al. reported the validity and reliability of the Persian version of BDI-II as acceptable within the Iranian population with high internal consistency (Cronbach’s alpha=0.87) and acceptable test-retest reliability (r=0.74). They also investigated the factor structure of the BDI-II-Persian, using confirmatory factor analysis (16).

#### 2.3.3 PQ

The Persian version of the PQ, recently translated and validated by Rafati et al., is an instrument designed to assess attitudes toward suicide in social crises. It presents 31 scenarios of social crises to participants. It assesses how likely participants think a typical person would consider suicide in each situation. Higher scores indicate higher levels of agreement with suicide as an option in crises. The Cronbach’s alpha for the Persian version of this questionnaire has been calculated as 0.94 (10).

### 2.4 Data management and analysis

After collecting the questionnaires, the data were entered into a secure electronic database using statistical software (e.g., SPSS). Each entry was cross-checked for accuracy to ensure data integrity. Missing data were handled through appropriate methods, such as imputation or exclusion, based on the specific analysis conducted and the nature of the missing information.

In addition to correlation analysis, we performed several statistical tests to comprehensively evaluate the data. Descriptive statistics were calculated to summarize the demographic and clinical characteristics of the participants. This included means, standard deviations, frequencies, and percentages for categorical variables.

We conducted Pearson’s correlation analysis to assess the relationships between the scores from the GHQ-28, BDI-II, and PQ questionnaires. The choice of Pearson’s correlation was based on the continuous nature of the data and the assumption of a linear relationship between the variables. The regression analysis examined the relationship between participants’ attitudes toward suicide (PQ score) and key predictors: GHQ-28 (mental health), BDI-II (depression), age, and gender.

## 3 Results

Out of 116 eligible participants sampled with Census methods, 85 accepted to enter our survey. The participants mentioned some reasons for their rejection in participation: some mentioned they were too stressed about the results of their session that they were not able to concentrate, others said the questionnaires were too long and they did not have enough time and some others were afraid that their responses influence the result of their session (although we emphasized on the confidentiality) and we respected their autonomy. We also excluded forms that were not completely or correctly answered. At last, we collected 78 forms (participation rate: 67.2%). Of these, 67.9% were assigned female-sex at birth. No participants identified outside the gender binary. This was in line with expectations for a GD sample in Iran. The age of the patients ranged from 18 to 38 years, with a mean age of 24.82 years (SD: 4.51). A total of 42.3% (33 out of 78) of the participants reported not having strong religious beliefs among all the participants, only six (7.7 percent) had a history of marriage to a person of a different biological sex. The demographic characteristics of the participants are provided in **Table 1**.

**Table 1:** Demographic characteristics of the participants.

Variable		N (%)
Assigned sex at birth	Male	25 (32.1%)
	Female	53 (67.9%)
Religiosity	No	37 (47.4%)
	Slight	26 (33.3%)
	Moderate	12 (15.4%)
	Strong	3 (3.8%)
Religion	Islam	45 (57.7%)
	Other religions	0 (0%)
	Not religious	33 (42.3%)
A history of marriage to a person of a different biological sex	Single	69 (87.3%)
	Married	9 (11.3%)

### 3.1 Mental health issues

Among all participants, the highest mean score was recorded in the social dysfunction subscale (8.02 ± 3.58), while the lowest mean score was in the depression subscale (5.24 ± 5.80). The total mean score was 24.98 (SD: 15.11), and according to the tool’s cut-off, 43 participants (55.1%) were identified as having mental health problems (**Table 2**).

**Table 2:** GHQ-28 subscales scores.

Variable	Mean	SD
Somatic Symptoms	5.79	4.09
Anxiety and Insomnia	5.92	4.40
Social Dysfunction	8.02	3.58
Depression	5.24	5.80
Total	24.98	15.11

No significant correlation was observed between the GHQ-28 total score and age or birth-assigned sex (p-value = 0.529, r= 0.072, and p-value = 0.857, t= 0.181, respectively).

### 3.2 Depression

The mean score for BDI-II was 9.84 (SD: 10.02) across all participants. Among these participants 32 (41.0%) had no signs of depression, 10 had minor depression, 15 were mildly depressed and 21 (26.9%) showed severe depression. No significant correlation was seen between age or sex and BDI-II scores (respectively p-value = 0.500, r= 0.078 and p-value = 0.870, t= 0.165).

### 3.3 Attitude toward suicide

The mean PQ score for participants was 57.92 (SD: 20.44). No significant correlation was observed between age or sex and PQ scores (respectively p-value = 0.593, r= 0.062 and p-value = 0.503, t= 0.673). Question number 21 *(Person L is a great fan of Person M, who is a popular singer, actor, and talk-show celebrity. Person M dies when a building collapses. Would person L have suicidal thoughts)?* received 71 “No” answers out of 78 participants, making it the item with the lowest level of endorsement considering attitudes toward suicide. Questions 8, 16, 29 (Question 8: *Person E suffers spinal injuries and will be confined to a wheelchair for life. Would person E have suicidal thoughts?*; Question 16: *Person J dropped a gas bottle which exploded. Person J sustained severe burns to the face and hands, which left disfiguring scars. Would person J have suicidal thoughts?*; Question 29: *Person U is convicted of rape and murder, and has been sentenced to life in jail without parole. Would person U have suicidal thoughts*)*?* received 17 “very much” answers. These items received the highest level of endorsement considering attitudes toward suicide among participants.

### 3.4 Association between attitude towards suicide and mental health of individuals with GD

There was a statistically significant correlation between the total score of GHQ-28 and PQ (r= 0.732 and p-value < 0.05). Also, a significant correlation was observed between BDI-II total score and PQ (r= 0.763 and p-value < 0.05).

The regression model, with an R-squared of 0.240, indicates that age, gender, GHQ-28, and BDI-II could explain 24% of the variance in attitudes toward suicide. Age was a significant predictor, while gender showed no significant effect. GHQ-28 and BDI-II scores were significant predictors of PQ, confirming a strong association between more severe mental health challenges and increased suicide-related attitudes (p < 0.05 for both variables).

## 4 Discussion

This study aimed to explore the attitudes toward suicide among individuals with GD, and their correlation with their depression and overall mental health.

A striking 67.9% of the participants had been designated female-sex at birth, showcasing a trend where assigned female rates surpass those of assigned males, a notable departure from Western statistics (6). Bayani et al. reported quality of life in assigned women at birth with GD in Iran after surgical treatment increased more than designated men at birth with GD, especially in aspects such as emotions, physical perfection, social desirability, and dependence (17). Since gender differences were not investigated in this study, it can be suggested for future studies.

Our study revealed a significantly higher prevalence of mental health issues among participants compared to the 23.44% reported in the last national survey conducted by Noorbala et al. in Iran (18). The mean score for BDI-II was 9.84 (SD: 10.02). When assessing depression levels using the BDI-II tool, a substantial 59% of participants exhibited varying degrees of depression. Interestingly, no discernible correlation emerged between age, gender, and depression scores within the cohort. Uyar et al. conducted a study on depression and anxiety in men with GD in Turkey, comparing their findings with a control group (19). They reported a BDI score of 12.97 (SD: 6.65) for their case group, closely mirroring the depression scores we observed in our participants. This correlation could potentially be attributed to cultural similarities between Iran and Turkey. Olson et al. reported, in a study at a large, urban transgender youth clinic in the United States, that 25 percent of the population with GD express symptoms of clinical depression (20), which was in line with our findings. Mahmoudi et al. (2019) delved into the realms of depression and suicidal ideation within a cohort of 60 individuals grappling with GD in Iran (21). The study unveiled a mean BDI-II score of 21.10 (SD: 14.16) among pre-surgery participants, a figure substantially higher than what manifested in our own research. This disparity could potentially be linked to the advanced stage of gender affirmation that characterized our study’s subjects, imbuing them with a sense of optimism. Noteworthy is the fact that our study encompassed a larger sample size post-surgery, a deliberate effort to surmount the limitations posed by the relatively diminutive sample size of 30 individuals in the pre-surgery phase addressed by the prior study.

The mean score of PQ was 57.92 (SD: 20.44). There was no significant correlation between age or gender and attitudes toward suicide. Unfortunately, as PQ is a newly introduced questionnaire, comparable studies are limited. Furthermore, there is no defined cut-off available to interpret the mean score. Based on our results, three questions (8, 16 and 29) describe situations with no cure or escape, received the highest endorsement regarding attitude toward suicide. People with GD consider incurable diseases such as spinal injury or disfiguring scars or situations with no escape like jail without parole, the most traumatizing. They may believe people in described situations are sentenced to live in pain and therefore suicide would be a good idea. On the contrary, question 21 *(Person L is a great fan of Person M, who is a popular singer, actor, and talk-show celebrity. Person M dies when a building collapses. Would person L have suicidal thoughts)?* received the lowest level of endorsement regarding attitudes toward suicide. According to Islamic beliefs, suicide is considered a grave sin and those who engage in it are believed to be condemned to hell. This strong conviction serves as a significant deterrent for Muslims and individuals raised within this culture against contemplating suicide. Moreover, Muslims firmly hold the belief in an eternal afterlife, which means death is not perceived as an absolute termination. Additionally, it is worth noting that, within Iranian culture, idolizing celebrities is not as prevalent as in other countries like the United States. Therefore, individuals in Iran may be less inclined to consider suicide as a viable course of action in response to challenging circumstances involving public figures (22).

Our study brought to light a significant and noteworthy discovery - a positive correlation between the total scores of the GHQ and BDI-II with PQ. This finding unveils a compelling link, suggesting that individuals with GD facing higher psychological distress levels, as indicated by elevated GHQ scores, tend to exhibit a more positive stance towards suicide. We argue that individuals experiencing more psychological distress are more exposed to suicidal ideation following a specific turn of events. They also tend to have less strength in facing these suicidal thoughts. Thus, not only the individuals with GD are more vulnerable under normal circumstances, but also, they are even more at risk of attempting suicide facing specific occasions.

A poignant observation emerges within the context of Iranian societal norms - despite prevalent negative perceptions towards individuals with GD and a dearth of adequate cultural and social support systems, many individuals from this community grapple with depression, cling to affirming views about suicide, and often navigate the absence of familial and societal backing. To navigate through this challenging landscape, it becomes imperative to advocate for the deployment of diverse psychological interventions such as individual, group, and family therapies, coupled with a heightened focus on bolstering societal understanding and acceptance of these individuals.

To the best of our knowledge, this is the first study to investigate attitudes toward suicide among individuals with GD. However, it is subjected to several limitations. These include its cross-sectional design, the small sample size, the fact that it was conducted at a single center, the absence of a suicidal ideation questionnaire, and using screening tools instead of confirmatory diagnosis. Additionally, the lack of a defined cut-off for the PQ further limits its applicability. Consequently, the generalizability of the study’s findings may be uncertain. Another limitation was the exclusive inclusion of patients in the final stages of gender-affirming surgery. We also indicate that our sample was predominantly assigned female-sex at birth. For future studies, we recommend considering larger sample sizes, a control group, multi-center studies, and varied study designs. Additionally, we suggest a comparative analysis of attitudes toward suicide before and after gender-affirming surgery.

## 5 Conclusions

Our study demonstrated that individuals experiencing greater psychological distress were more likely to hold a favorable attitude toward suicide. Additionally, the high prevalence of suspected mental health issues within this population underscores the profound psychological burden faced by individuals with GD. As a group already identified as highly vulnerable to suicide, those with GD endure significant emotional and mental strain, which only amplifies their susceptibility to suicidal ideation. These findings highlight the urgent need for tailored mental health interventions and underscore the importance of integrating comprehensive psychological care into healthcare policies. Addressing the mental health needs of individuals with GD is not only a clinical imperative but also a critical step in reducing the broader risk of suicide in this population. By prioritizing their mental well-being, we can help alleviate the severe distress they endure and improve their overall quality of life.

## Data Availability

The raw data supporting the conclusions of this article will be made available by the authors, without undue reservation.
